# Gene supplementation therapy restores motor deficits in Gnao1[C215Y] mice and proves broad efficacy in *C. elegans* model

**DOI:** 10.1186/s40478-026-02389-6

**Published:** 2026-07-21

**Authors:** Germana Cocozza, Tiziano D’Andrea, Martina Di Rocco, Ludovica Maria Busdraghi, Gilda Chilà, Xingzi Lin, Enrico Lanza, Fanny Collaud, Francesca C. Follo, Clelia Buccheri, Viola Folli, Giuseppe Ronzitti, Ferdinando Scavizzi, Marcello Raspa, Vincenzo Leuzzi, Vladimir L. Katanaev, Serena Galosi, Sergio Fucile, Simone Martinelli, Giuseppina D’Alessandro, Cristina Limatola

**Affiliations:** 1 Department of Physiology and Pharmacology, Sapienza University of Rome, Rome, Italy; 2 IRCCS Neuromed, Pozzilli, Italy; 3 Department of Oncology and Molecular Medicine, Istituto Superiore di Sanità, Rome, Italy; 4 D-tails, Rome, Italy; 5 Université Paris-Saclay, University Evry, Integrare Research Unit UMR_S951, Inserm, Genethon, Evry, 91000 France; 6 Genethon, Evry, 91000 France; 7 National Research Council (Institute of Biochemistry and Cell Biology), Campus International Development (European Mouse Mutant Archive- INFRAFRONTIER), Monterotondo Scalo, Rome, Italy; 8 Department of Human Neuroscience, Sapienza University of Rome, Rome, Italy; 9 Department of Cell Physiology and Metabolism, Faculty of Medicine, Translational Research Center in Oncohaematology, University of Geneva, Geneva, 1211 Switzerland; 10 Qatar Biomedical Research Institute, College of Health and Life Sciences, Hamad Bin Khalifa University, P.O. Box 34110, Doha, Qatar; 11 Laboratory affiliated to Istituto Pasteur Rome, Department of Physiology and Pharmacology, Sapienza University of Rome, Rome, Italy

**Keywords:** *GNAO1*, Gαo, Gene therapy, Behavioral tests, Mouse model, Electrophysiological recordings, *C. elegans*

## Abstract

The heterotrimeric G proteins are ubiquitous membrane-bound complexes specialized in the transduction of receptor-mediated extracellular signals into intracellular responses. Mutations in the G protein subunit alpha O1 (Gαo), encoded by *GNAO1*, have been associated with neurodevelopmental disorders characterized by prominent movement disorder with or without epilepsy. The *Gnao1*[C215Y]/+ mouse model recapitulates key features of the human disease, with a relatively mild phenotype, normal viability and late onset movement abnormalities, detectable in specific motor tasks. Here we report functional alterations in cortical layer V pyramidal neurons from *Gnao1*[C215Y]/+ mice, with reduction of the inward currents (Ih) and impairment of the GABAB-mediated outward current. We propose an adeno-associated virus (AAV)-based gene therapy aimed at overexpressing the wild-type form of Gαo by intracerebroventricular injection in newborn heterozygous *Gnao1*[C215Y]/ + mice. Our results demonstrate that overexpression of the wild-type protein mitigates behavioral abnormalities and restores functional neuronal deficits in young adult mice, thereby supporting the development of AAV-mediated gene augmentation strategies to counteract the effects of *GNAO1* variants in patients. The potential of gene supplementation therapy is further supported by data from genetically modified *C. elegans* strains carrying not only the relatively mild C215Y variant but also a panel of *goa-1*/*GNAO1* mutations associated with more severe phenotypes, suggesting a broader potential of this therapeutic strategy.

**Supplementary Information:**

The online version contains supplementary material available at https://doi.org/10.1186/s40478-026-02389-6.

## Introduction

De novo mutations in GNAO1, which encodes the abundant neuronal G protein Gαo, have been associated with a broad spectrum of neurological disorders [1–5] ranging from early-infantile severe developmental epileptic encephalopathy (DEE17) [5] to late-infantile early childhood neurodevelopmental stagnation or regression and dyskinetic movements as prevalent features [1–4, 6, 7] to milder movement disorder phenotypes, typically dystonia-parkinsonism with or without mild cognitive impairment. These phenotypes are substantially stable or show slow progression from late childhood to adulthood [8].

To date, approximately 250 patients with pathogenic variants in the GNAO1 gene have been described, and it is estimated that around 400 patients worldwide are affected by this severe and underdiagnosed condition. In most patients, GNAO1-related Movement disorders (MD) is extremely disabling, with a clinical signature that includes mixed dyskinesias such as dystonia, chorea, stereotypies, and parkinsonism, as well as life-threatening dyskinetic crises triggered by a wide range of factors, including excitement/emotion, fever, high external temperature, infections, intentional movements, and the sleep/wake cycle [7].

Currently, no pharmacological approach provides complete treatment for severe GNAO1-related MDs. Zinc and caffeine are under investigation as potential treatments for the control of GNAO1-related hyperkinetic MDs [9–12]. Globus pallidus internus deep brain stimulation (GPi-DBS), an invasive surgical procedure, is considered the only available therapeutic option for adequately controlling paroxysmal episodes [7], but it is only minimally effective in treating the fixed severe MDs associated with this condition [13, 14].

Among *GNAO1* pathogenic variants, the c.644G > A nucleotide substitution, which is predicted to cause the p.C215Y amino acid change, is associated with a relatively late age of onset (3 to 12 years), no signs of epilepsy, moderate or absent intellectual disability, and generalized dystonia, enabling affected individuals to lead fulfilling lives compared to other patients with more frequent and severe *GNAO1* variants [8]. Accordingly, knock-in mice heterozygous for the C215Y variant exhibit hyperlocomotion detectable in a subset of behavioral tests [15]. These motor alterations mirror the symptoms observed in patients harboring this variant [8, 16]. Mice also show reduced numbers of neuronal precursors and dilated lateral ventricles [15]. A growing body of preclinical evidence suggests that AAV vectors are powerful tools for gene therapy, and AAV9 is widely used as a capsid in gene therapy-based clinical trials [17]. A recent report describes the use of an AAV-RNAi strategy to silence a dominant variant of Gnao1 in mice, with partial functional rescue in neurons [18].

We aimed to test the hypothesis that overexpressing the wild-type GNAO1 gene could counteract the functional alterations associated with the C215Y variant in mice.

To this end, we employed two widely used animal models for investigating human diseases: mice and the nematode Caenorhabditis elegans (*C. elegans*). Treatment with an AAV carrying the wild-type GNAO1 allele in newborn C215Y mutant mice attenuated the functional alterations caused by the mutant protein. The treated mice exhibited less anxiety-like behaviour, with motor performance comparable to that of wild-type mice. Furthermore, this study expanded our understanding of C215Y-related neuronal alterations in mice, including changes in resting membrane potential and G-protein-activated currents, particularly those mediated by GABA-B receptor activation.

The relevance of *C. elegans* as a model for GNAO1-related disorders has been well established [11–12, 19]. In *C. elegans*, Gαo, encoded by *goa-1*, the orthologue of *GNAO1*, negatively regulates synaptic vesicle release in HSN and ventral cord motor neurons, thereby modulating egg-laying and locomotion, respectively [20]. Using CRISPR-Cas technologies, we generated and characterized *C. elegans* strains carrying a panel of pathogenic *goa-1* variants. Each variant produced a distinct pattern of locomotor defects, reflecting the phenotypic heterogeneity observed in patients. In this study, we investigated the functional impact of the C215Y *GNAO1* variant in *C. elegans* and performed a proof-of-concept study to evaluate gene supplementation as a therapeutic strategy for GNAO1 encephalopathy, independently of the specific mutation involved.

Collectively, the data reported in this study support the potential of AAV-mediated gene augmentation as a therapeutic strategy to counteract the pathogenic effects of *GNAO1* variants in patients.

## Methods

### Animal treatment and surgery

Experiments described in the present work were approved by the Italian Ministry of Health (authorization n° 663/2023-PR) in accordance with the guidelines on the ethical use of animals from the European Community Council Directive of 22 September 2010 (2010/63/EU), and from the Italian D.Lgs 26/2014. Male and female *Gnao1*[C215Y]/+ mice [15] were maintained at 20–24 °C and 45–65% humidity, with a 12/12 h light/dark cycle; water and food were provided *ad libitum*. Newborn (P0–P1) *Gnao1*[C215Y]/+ mice, obtained crossing *Gnao1*[C215Y]/[C215Y] and Gnao1+/+ mice, underwent bilateral intracerebroventricular (ICV) injections into the anterior horn of the lateral ventricles. The wild-type human GNAO1 coding sequence (hGNAO1; NCBI RefSeq NM_020988.3, nucleotides 748–1812) was synthesized de novo (GeneCust, Luxembourg). The hGNAO1 transgene or the reporter gene Emerald Green Fluorescent Protein (EmGFP) was cloned into an AAV expression cassette under the control of the ubiquitous CAG promoter. The expression cassette additionally contained the woodchuck hepatitis virus post-transcriptional regulatory element (WPRE) and the human growth hormone polyadenylation signal (hGH polyA) to enhance transgene expression and mRNA stability. The entire cassette was flanked by AAV2 inverted terminal repeats (ITRs) to allow vector genome packaging. Recombinant AAV particles were pseudotyped with the AAV9 capsid and produced by an adenovirus-free triple-transfection protocol in suspension-adapted HEK293 cells. Viral vectors were purified by affinity chromatography as previously described (PMID: 30705921). Vector genome titers were quantified by real-time quantitative PCR (qPCR) using primers targeting the AAV2 ITR sequence (forward: 5′-GGA ACC CCT AGT GAT GGA GTT-3′; reverse: 5′-CGG CCT CAG TGA GCG A-3′). Titers were expressed as vector genomes per milliliter (vg/mL). Pups were divided in six groups based on genotype and type of treatment: AAV9.CAG.GFP-injected wt mice; AAV9.CAG.*hGNAO1*-injected wt mice, AAV9.CAG.GFP-injected heterozygous mice; AAV9.CAG.*hGNAO1*-injected heterozygous mice. Mice were anesthetized and stereotaxically injected using a 33-gauge stainless steel Hamilton syringe. Each animal received 5 µL of viral vector (1 × 10⁸ viral genomes/µL) via intracerebroventricular (ICV) injection. Following injection, pups were returned to the dam and maintained with the litter until weaning. At postnatal day 21, mice were weaned and group-housed (2–3 animals per cage) until use in subsequent experiments.

### Western Blot analysis

Total proteins were extracted from brain tissues in RIPA buffer, followed by quantification using Pierce™ BCA Protein Assay Kit (Thermo Scientific, Cat# 23227) and separation by SDS-PAGE. After blocking with non-fat dry milk at room temperature, the membrane was incubated overnight at 4 °C with primary antibody anti-GNAO1 (1/1000; Abcam, #EPR24151-69). Subsequently, the HRP-conjugated secondary antibody (1/2000; Agilent Technologies, Cat# P0448; RRID: AB_2617138) was incubated with the membrane (1 h, room temperature). Blots were visualized using Clarity™ Western ECL Substrate (Bio-Rad, Cat#170–5061). β-actin (Sigma-Aldrich, Cat# A2228; RRID: AB_476697) was selected as an internal reference.

### Behavioral tests

Experimental groups were blinded and randomly assigned before the start of behavioral experiments and remained blinded until all data were collected. Mice were tested at 10–12 weeks of age. Sample sizes were chosen on the basis of a power analysis using estimates from previously published experiments. All animals were handled for at least 5 min/day for 2–3 days before starting the experiments. Before all experiments, mice were transported to the behavior room and left at least 30 min to habituate.

#### Open-field test

This test is used to assess general locomotor activity levels, anxiety-like behavior, and willingness to explore. Individual mice were placed in the corner of an enclosed platform (40 cm× 40 cm× 30 cm). The total distance traveled, velocity, and time spent in the center area (20 cm× 20 cm) were recorded for 10 min and automatically scored with ANYMAZE 7.0 (Ugo Basile, Italy) software.

#### Rotarod test

Motor coordination, strength and balance were assessed using a rotarod apparatus (Ugo Basile, Gemonio Italy, #47650). Mice were subjected to two trials per day for four consecutive days with 20-min intervals between trials. The animals were placed on the cylinder at an accelerated rotarod speed that increased from five rotations per minute (RPM) to 40 RPM, the arbitrary cut-off time was 300 s., and the longest latency was recorded.

### Electrophysiological recordings on cortical neurons in mice

Whole-cell patch-clamp recordings were performed on cortical layer V pyramidal neurons from all experimental groups between 19 and 21 weeks of age. Membrane currents and potentials were recorded by using glass electrodes (3–4 MΩ) filled with 140 mM KGluconate, 10 mM Hepes, 5 mM BAPTA, and 2 mM Mg-ATP (pH 7.3, with KOH). Considering that experiments did not focus on Ca^2+^-dependent processes, BAPTA 5 mM was used to improve whole-cell recording stability and preserve patch health, as well to prevent high [Ca^2+^]_i_ able to inhibit GIRK-mediated currents [21]. During recording, cells were continuously superfused using a gravity-driven perfusion system where perfusion tubes were connected to a fast exchanger system (RSC-160; Bio-logic, Claix, France). The aCSF (artificial Cerebrospinal Fluid) was composed of (in mM): 125 NaCl, 3 KCl, 26 NaHCO_3_, 1.25 NaH_2_PO_4_, 2 CaCl_2_, 1 MgCl_2_, and 25 glucose; pH value adjusted to pH 7.3 with NaOH 1 M. The identification of neurons followed morphological criteria: high-diameter cells with a triangular shape. Evoked action potentials (AP) were recorded in current-clamp using 1 s current steps (5–10 pA, 12 steps). Ih currents were measured by clamping the membrane potential at − 80 mV, − 100 mV and − 120 mV (duration 1 s). We also recorded synaptic activity in voltage-clamp at − 70 mV for 15 min; after 5 min, using perfusion tubes, baclofen 100 µM was delivered for 5 min to observe GABA_B_-mediated outward current. Membrane potentials and currents were acquired at 50 kHz and filtered at 3 kHz with an amplifier HEKA EPC 800 (HEKA Elektronik, Reutlingen, Germany), a Digidata 1322 A analog-to-digital converter, and pClamp 10 software (Molecular Devices, Union City, CA), and analyzed off-line.

Statistical differences were assessed using SigmaPlot 16.0 software. All data were expressed as means ± S.D. and analysed using one-way ANOVA or two-way ANOVA, as appropriate. Tests for normality and variance heterogeneity were conducted on each data set. When necessary, the non-parametric Dunn’s one-way ANOVA on ranks was used. In case of significance, all pairwise multiple comparison procedures were used (Holm-Sidak or Dunn’s method for non-parametric tests). The minimum power of statistical tests was set at 0.8. The significance for all tests was set at *p* < 0.05. The two-way ANOVA was performed to analyze the effect of the C215Y mutation (factor A) and h*GNAO1* insertion (factor B) on the dependent variable in the operated mouse groups.

### *C. elegans* studies

*C. elegans* strains were maintained at 20 °C on nematode growth medium (NGM) seeded with OP50 E. coli using standard techniques [22]. Microinjections and genetic crosses were performed as described [23, 24]. The Bristol N2 (control) and the DG1856 *goa-1(sa734)* null strains were provided by the *Caenorhabditis* Genetic Center (University of Minnesota, Minneapolis, MN). The generation of the PHX7956 *goa-1(syb7956)* strain carrying the homozygous C215Y variant was outsourced (Fujian SunyBiotech Co., Fuzhou, China).

Synaptic vesicles release by ventral cord motor neurons was evaluated by aldicarb assay. Aldicarb sensitivity was assessed in young adult hermaphrodites from synchronized cultures, following the method described by Mahoney et al. 2006 [25]. Briefly, worms were placed on NGM plates containing 500 µM aldicarb (Sigma-Aldrich, Saint Louis, MO) and monitored at 10 min intervals. Paralysis was determined by the absence of movements in response to gentle touch with an eyelash. Phenotypic observations were performed using a Nikon SMZ18 stereomicroscope (Nikon Europe, Amstelveen, NL). Data were expressed as the percentage of animals that remained motile at each time point. Due to variability between aldicarb batches, comparisons were considered valid only among lines/strains tested within the same experimental set.

Synaptic vesicles release by HSN motor neurons was assessed by counting the eggs retained in the uterus of adult hermaphrodites during the exponential egg-laying phase. Observations were performed on live animals mounted on 2% agarose pads containing 10 mM sodium azide as anesthetic, using a SMZ18 stereomicroscope and an Eclipse Ti2-E microscope equipped with Nomarski optics (Nikon Europe, Amstelveen, NL).

Locomotion parameters (reversal rate, body bends, and speed) were analyzed on synchronized L3 larvae using a recently developed automated tracking system [25]. Videos were generated at 10 frames/sec for 2 min and were processed using a customized MATLAB software (Mathworks, Massachusetts, MA). Locomotion speed was calculated by measuring the distance travelled between consecutive frames, based on the position of the worm’s centroid along a consistent trajectory. Reversals are defined as backward movements covering a distance at least equal to the length of the pharynx (approx. one-fifth of the animal’s total body length). Body bends are defined as directional changes in the worm’s mid-body curvature during locomotion, with each bend counted when curvature peaks in the opposite direction from the previous one.

For rescue experiments, the wild-type *goa-1* cDNA was amplified and cloned into the pBG-GY152 plasmid under the control of the pan-neuronal *rgef-1* promoter (a kind gift by B. Grill, Seattle, WA) and injected at a concentration of 30 ng/µL to generate multi-copy extrachromosomal arrays. Microinjections at a higher concentration (100 ng/µL) resulted in embryonic lethality. The pJM371 plasmid (p*WE WE-2*::*NLS*::*GFP*), a kind gift from J.D. McGhee (University of Calgary), was used as a co-injection marker (30 ng/µL), enabling GFP expression in intestinal cell nuclei. Transgenic lines overexpressing the wild-type *goa-1* allele in *C. elegans* neurons were crossed with genetically modified strains for rescue experiments. For each genotype, three independent clones were isolated and evaluated phenotypically.

Statistical differences were assessed using GraphPad Prism 8.4.2 software. Locomotor parameters were analyzed through one-way ANOVA with Bonferroni correction for multiple comparisons. The log-rank test was used for Kaplan Meier survival curves (aldicarb sensitivity). To reduce risks of bias, genotype blinding and randomization were applied during testing and analyses. Isogenic animals were cloned separately and used as controls in all experiments.

## Results

### Expression of Human Gαo

**Fig. 1:**
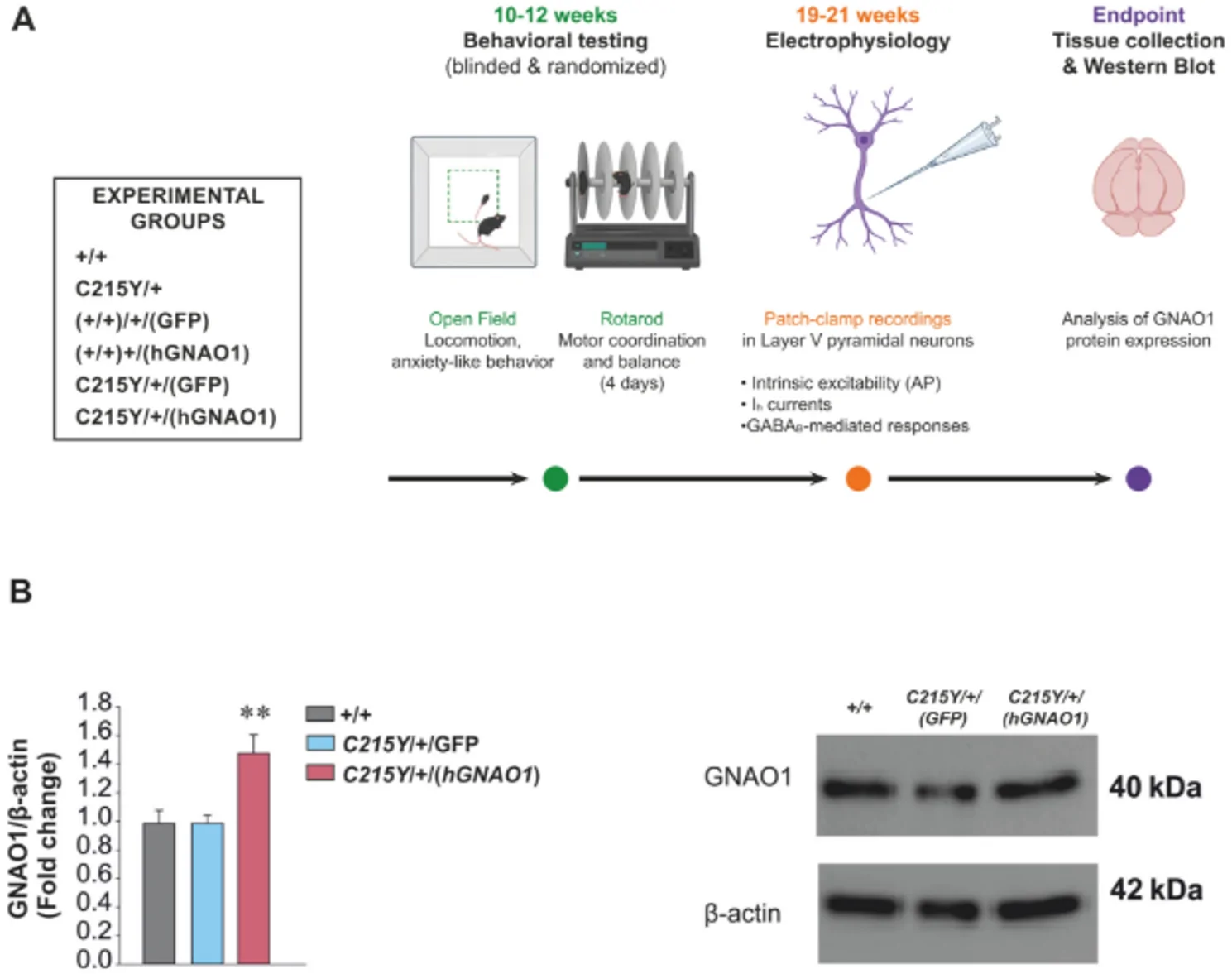
**A** Experimental design and study timeline. Gnao1*[C215Y]*/+ mice received bilateral intracerebroventricular (ICV) AAV9.CAG.GFP or AAV9.CAG.hGNAO1 injections at P0–P1. All groups underwent behavioral testing (open-field and rotarod) was performed between 10 and 12 weeks of age under blinded and randomized conditions. Electrophysiological recordings from cortical layer V pyramidal neurons were conducted between 19 and 21 weeks of age. At the experimental endpoint (> 22 weeks), brain tissue was collected for Western blot analysis of GNAO1 protein expression. **B** Quantification of GNAO1 normalized to β-actin and expressed as fold change relative to wild-type littermates (+/+). Data are shown as mean ± SEM; **p < 0.01 by one-way ANOVA. Representative Western blot showing GNAO1 protein levels in brain tissue from wild-type (+/+), *Gnao1[C215Y]*/+/(GFP), and *Gnao1[C215Y]*/+/(h*GNAO1*) mice (*N* = 3), with β-actin used as a loading control, is shown on the right

Here, we propose an AAV-based gene therapy to overexpress the wild-type human Gαo protein via intracerebroventricular injection in newborn heterozygous Gnao1[C215Y]/+ mice. Experimental design is shown in Fig. 1A. Figure S1 shows the efficiency of the AAV9.CAG.GFP administration protocol across different brain regions. Western blot analysis revealed that even at long time after injection (22 weeks), the amount of Gαo protein was significantly increased in Gnao1[C215Y]/+(hGNAO1) mice compared to both wild-type (+/+) and Gnao1[C215Y]/+(GFP) mice (Fig. 1B). We speculate that higher expression of GNAO1 could have been detected at earlier times, not analyzed for the specificity of experimental design. Full blots are shown as Fig. S2. For the subsequent experiments, mice were divided into six groups based on genotype and treatment: untreated wild-type mice (+/+); untreated heterozygous mice (*Gnao1*[C215Y]/+); AAV9.CAG.GFP-injected wild-type mice +/+/(GFP), AAV9.CAG.h*GNAO1*-injected wild-type mice +/+/(hGNAO1), AAV9.CAG.GFP-injected heterozygous mice (*Gnao1*[C215Y]/+(GFP)); AAV9.CAG.h*GNAO1*-injected heterozygous mice (*Gnao1*[C215Y]/+(h*GNAO1*).

### Behavioral alterations in Gnao1[C215Y]/+ mice and effects of AAV-mediated GNAO1 expression

**Fig. 2:**
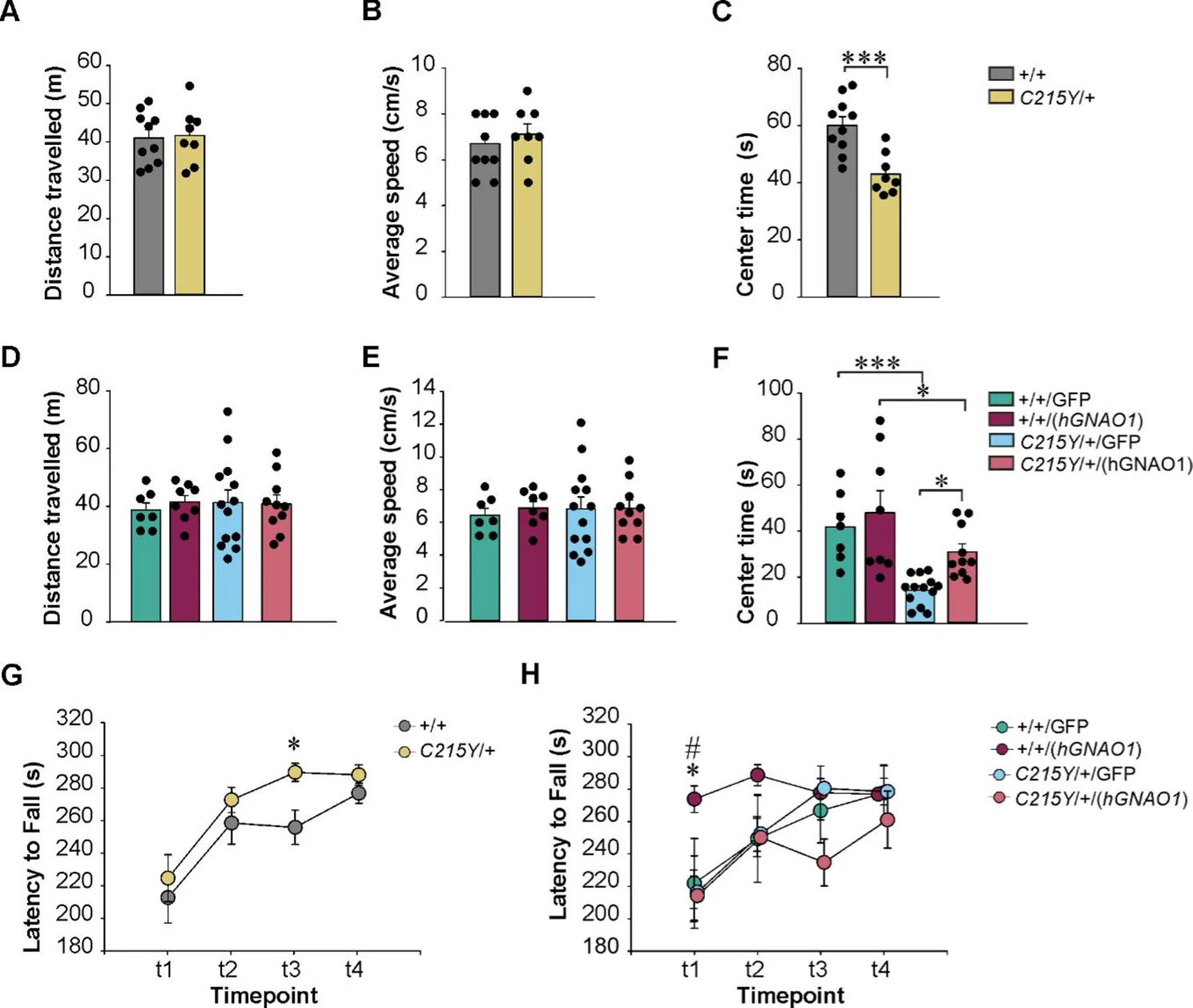
Open field performance scoring total distance travelled (**A**,** D**), average speed (**B**,** E**) and time spent in the center of the apparatus (**C**,** F**) on +/+ (*n* = 9), C215Y/+ (*n* = 9) mice and on +/+/(GFP) (*N* = 7), +/+/(h*GNAO1*) (*N* = 8), *Gnao1[C215Y]*/*+*/*(*GFP) (*N* = 13) and *Gnao1[C215Y]*/+/(hGNAO1) (*N* = 10) mice. Statistical analysis: one way (A-C) and two-way (D-F) ANOVA, *p < 0.05, ***p < 0.001; (**G**,** H**) Performance on accelerated rotarod (5 to 40 rpm) over four consecutive days scored on accelerated rotarod scored on (**G**) +/+ (*N* = 14), Gnao1[C215Y]/+ (*N* = 10); (**H**) +/+/(GFP) (*N* = 7) and +/+/(*hGNAO1*) (*N* = 8), Gnao1[C215Y]/+/(GFP) (*N* = 16) and Gnao1[C215Y]/+/(hGNAO1) (*N* = 11). Statistical analysis, one- (**G**) and two-way (**H**) ANOVA, +/+/(*hGNAO1*) vs. Gnao1[C215Y]/+/GFP #*p* < 0.05; +/+/(*hGNAO1*) vs. Gnao1[C215Y]/+/(hGNAO1) *p < 0.05

We chose two behavioral tests to characterize exploratory behavior and motor skills in mice carrying the C215Y variant and after AAV treatments. The open field was used to assess exploratory behavior and general locomotion in mice over 10 min. As shown in Fig. 2A and B, neither the total distance travelled nor the locomotor speed differed between wild-type (+/+) littermates and *Gnao1*[C215Y]/+ mice. However, Gnao1[C215Y]/+ mice spent significantly less time in the central area of the arena compared with wild-type animals, indicating increased anxiety-like behavior (Fig. 2C). To evaluate the effect of human GNAO1 expression, mice were transduced with either GFP or hGNAO1 constructs. No significant differences were observed among the four experimental groups [+/+/(GFP), +/+/(hGNAO1), C215Y/+/(GFP), and C215Y/+/(hGNAO1]) in total distance travelled or average speed, confirming that neither the mutation nor transgene expression affected general locomotor activity (Fig. 2D-E) whereas the time spent in the center was markedly affected (Fig. 2F). Consistently with not injected mice, C215Y/+/(GFP) mice spent significantly less time in the center than +/+/(GFP). The expression of hGNAO1 significantly increased the center time in C215Y/+ mice compared to C215Y/+/(GFP) mice, indicating a partial rescue of the anxiety-like phenotype, although the values remained lower than those observed in wt animals. No significant effect of hGNAO1 expression was detected in wt mice. An increase in anxiety-like behavior was observed in mice that underwent neonatal surgery compared to untreated mice, consistent with previous evidence linking neonatal intracranial interventions to altered emotional behavior later in life [26].

To evaluate motor coordination, a modified rotarod test was used [15] where the rotating speed was not constant, but accelerated during the test (from 5 to 40 rpm in 300 msec). As already reported, both wild-type (+/+) and C215Y/+ mice showed progressive improvement in performance across trials until t2, indicating motor learning over time (Fig. 2G). At t3, however, C215Y/+ mice outperformed their wt littermates, similar to what previously observed [15]. We have no explanation for the behavior of wt mice but we recorded these variations in 100% of tested mice. No differences were observed at later time points.

In AAV-treated mice (Fig. 2H), +/+/(hGNAO1) mice exhibited a significantly longer latency to fall at t1 compared to all the other experimental groups. No genotype-dependent differences were observed at later time points, although Gnao1[C215Y]/+(hGNAO1) mice performed similarly to wt mice at t3. Together, these data suggest that AAV-GNAO1 expression reduces anxiety-like behavior in Gnao1[C215Y]/+ mice, while its effects on motor performance require further investigation.

### Effects of AAV-mediated GNAO1 expression on the neuronal functional alterations of Gnao1[C215Y] mutant mice

**Fig. 3:**
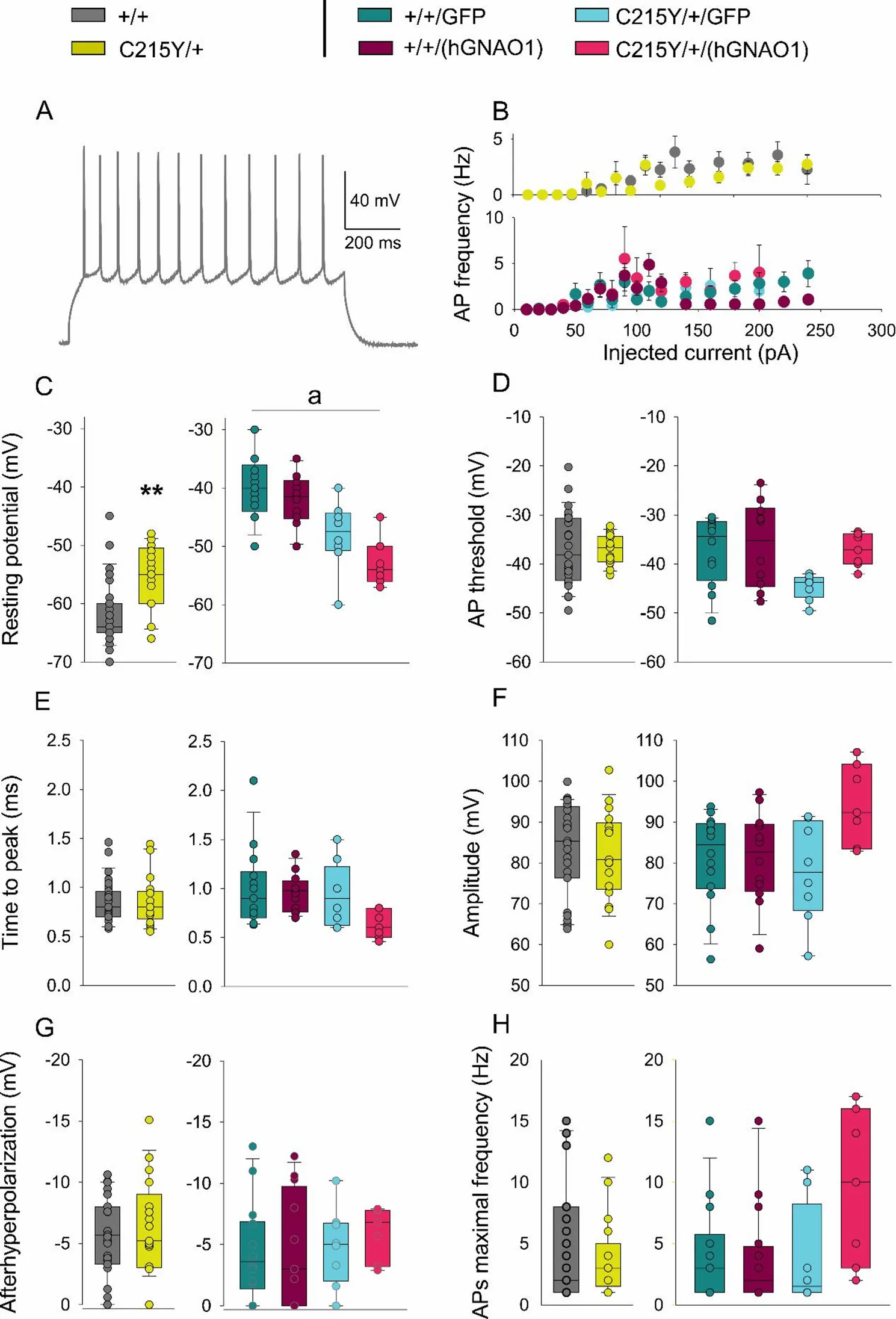
*C215Y Gnao1 mutation does not alter the properties of APs recorded from layer V pyramidal neurons in slices.* Evoked APs were recorded in the current clamp configuration from layer V of cortical pyramidal neurons. **A**, typical trace of an evoked AP train, from a +/+ neuron with 60 pA injected. **B** Input-output relationship between injected current and the number of elicited APs, for untreated mice (+/+, grey, and C215Y/+, yellow, top panel; N_mice_= 7 and 5, n_cells_= 28 and 18, respectively) and AAV-treated mice (+/+/GFP, green; +/+/(h*GNAO1*), dark red; C215Y/+/GFP, light blue; and C215Y/+/(h*GNAO1*), light red, bottom panel; N_mice_= 3 for all groups, n_cells_= 14, 10, 9 and 8, respectively); there is no statistical significance. Error bars correspond to standard deviations. **C** box plots of the resting potential of pyramidal neurons from untreated (left) and AAV-treated mice (right). A statistically significant difference is present between +/+ (mean = -62 ± 1 mV) and C215Y/+ (mean = -55 ± 1 mV, **: *p* < 0.001, one-way ANOVA), on the left graph; +/+/GFP (mean = -39 ± 1.5 mV), +/+/(h*GNAO1*) (mean = -42 ± 1.33 mV), C215Y/+/GFP (mean = -48 ± 2.1 mV) C215Y/+/(h*GNAO1*) (mean = -53 ± 1.5 mV), on the right graph, also are significantly different (two-way ANOVA, a: factor A (C215Y mutation), *p* < 0.001; factor B (h*GNAO1* insertion), *p* = 0.039) **D** box plot of AP thresholds from untreated (left) and treated mice (right). No significant differences. **E** box plot of AP times to peak from untreated (left) and treated mice (right). No significant differences. **F** box plot of AP amplitudes from untreated (left) and treated mice (right). No significant differences. **G** box plot of AP after hyperpolarization values from untreated (left) and treated mice (right). No significant differences. **H** box plot of AP maximal frequencies from untreated (left) and treated mice (right). No significant differences

To evaluate the functional outcome of the C215Y mutation as well as of the overexpression of the wild-type *GNAO1* allele in the mutant animals, we measured the functional parameters of action potentials (APs) evoked from layer V pyramidal neurons (Fig. 3A). The relationships between injected current and APs frequency showed no differences (Fig. 3B). The C215Y mutation caused more depolarized resting membrane potential in the untreated mice neurons (Fig. 3C, left), while in the AAV-treated mice a basal neuronal depolarization was observed, which was reduced in the group treated with the (h*GNAO1*) insertion (Fig. 3C, right). The mutation, however, did not significantly affect the functional properties of evoked APs and adenovirus treatment had no obvious effect (Fig. 3D–H).

**Fig. 4:**
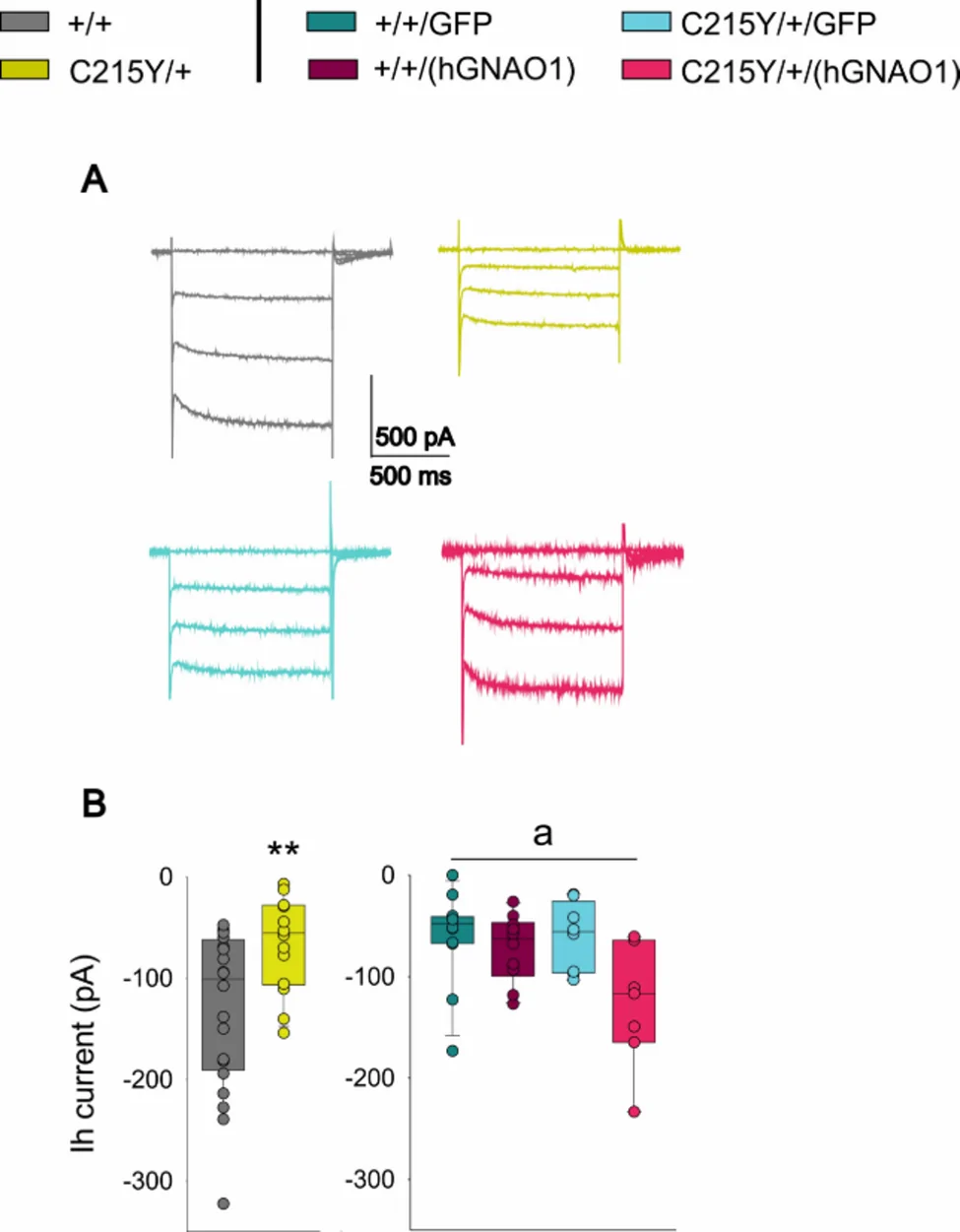
*C215Y Gnao1 mutant mice show a reduction of the Ih current*,* while hGNAO1-infected mice show a restoration to a pre-operation (physiological) condition.***A** some typical traces of evoked Ih current for the experimental groups: +/+ (grey, upper left), C215Y/+ (yellow, upper right), C215Y/+/(GFP) (light blue, lower left) and C215Y/+/(h*GNAO1*) (light red, lower right). Ih currents were evoked by hyperpolarizing the neurons with voltage steps (from − 60 mV to − 80 mV, − 100 mV and − 120 mV) in voltage-clamp configuration. **B** amplitudes of the Ih currents from untreated (left panel) and AAV-treated mice (right panel) at − 120 mV; there are statistically significant differences: +/+ (mean = − 132 ± 18 pA) differs from C215Y/+ (mean = − 66 ± 12 pA, a: **p = 0.009, one-way ANOVA; N_mice_= 7 and 5, n_cells_= 21 and 15, respectively) on the left panel; +/+/GFP (mean = − 60 ± 13 pA), +/+/(h*GNAO1*) (mean = − 72 ± 11 pA), C215Y/+/GFP (mean = − 61 ± 12 pA) C215Y/+/(h*GNAO1*) (mean = − 129 ± 23 pA), on the right graph, also are significantly different (two-way ANOVA, a: factor B (h*GNAO1* insertion), *p* = 0.011; N_mice_= 3 for all groups, n_cells_= 12, 12, 8 and 7, respectively), showing a functional rescue

In the same neurons, we also recorded the Ih current, consequent to the activation of hyperpolarization-activated HCN channels (Fig. 4A). Interestingly, at − 120 mV, the C215Y mutation was associated with a significant reduction of the Ih current amplitude in the untreated mice (Fig. 4B, left panel), while the overexpression of the wild-type *GNAO1* construct rescued the Ih current function (Fig. 4B, right panel).

**Fig. 5:**
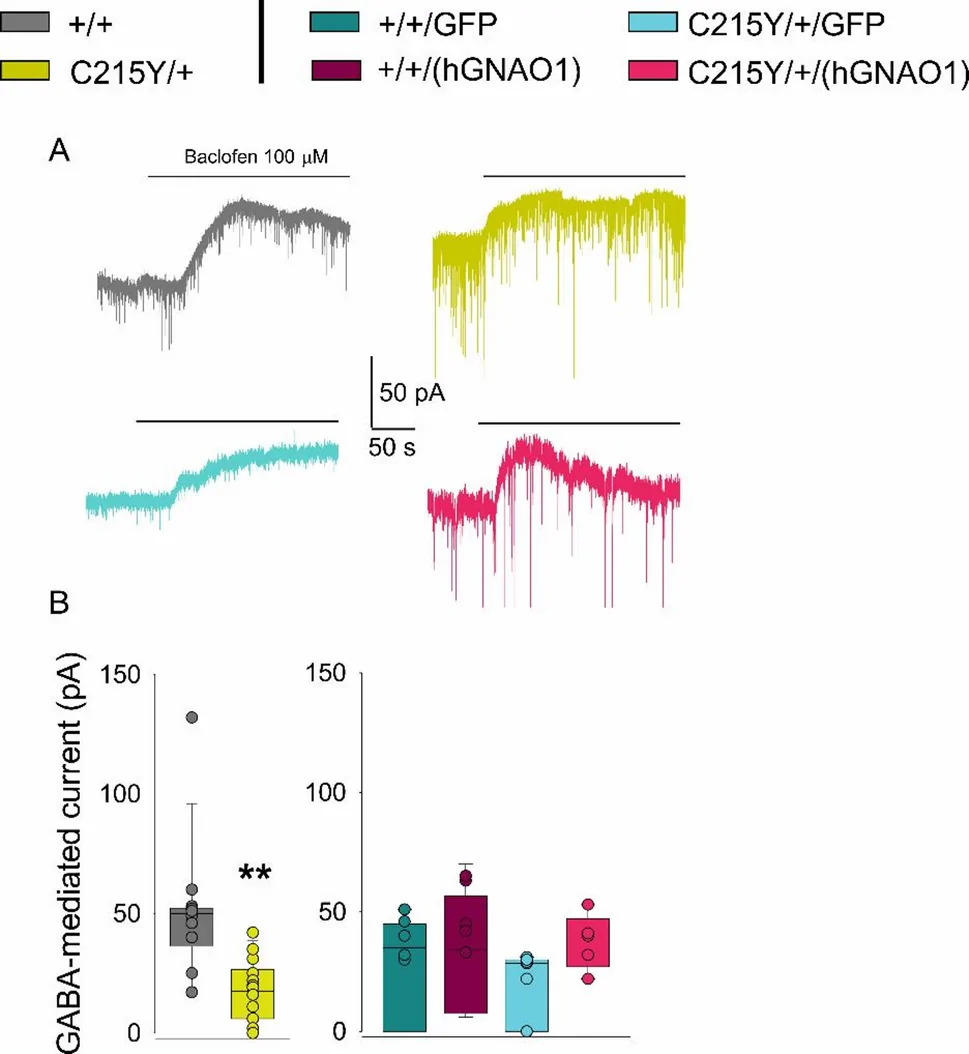
*C215Y Gnao1 mutation causes an impairment of the GABA*_*B*_*-mediated outward current.***A** some typical traces recorded in voltage-clamp configuration from neurons of the four experimental groups: +/+ (grey, upper left), C215Y/+ (yellow, upper right), C215Y/+(GFP) (light blue, lower left) and C215Y/+(h*GNAO1*) (light red, lower right). Spontaneous post-synaptic currents are visible, as well the slow outward currents evoked by the application of the selective GABA_B_ receptor agonist baclofen (5 min, 100 µM). **B** amplitudes of the baclofen-evoked outward current from untreated (left panel) and AAV-treated mice (right panel): +/+ (mean = 50 ± 7 pA) differs from C215Y/+ (mean = 18 ± 3 pA, ** *p* < 0.001, one-way ANOVA; N_mice_= 7 and 5, n_cells_= 14 and 14, respectively) in the left panel; there are no statistically significant differences in AAV treated mice, in the right panel (N_mice_= 3 for all groups, n_cells_= 6, 7, 7 and 6)

Last, we measured the amplitude of the slow outward currents evoked by the application of the selective GABA_B_ receptor agonist baclofen (Fig. 5A). The C215Y mutation was associated with a significant reduction of these G-protein-activated outward currents in untreated mice (Fig. 5B, left panel), which was maintained in AAV-treated mice, without significant effects upon h*GNAO1* expression (Fig. 5B, right panel).

### Overexpression of wild-type goa-1 in C. elegans neurons improves the phenotype of goa-1[C215Y] animals

**Fig. 6:**
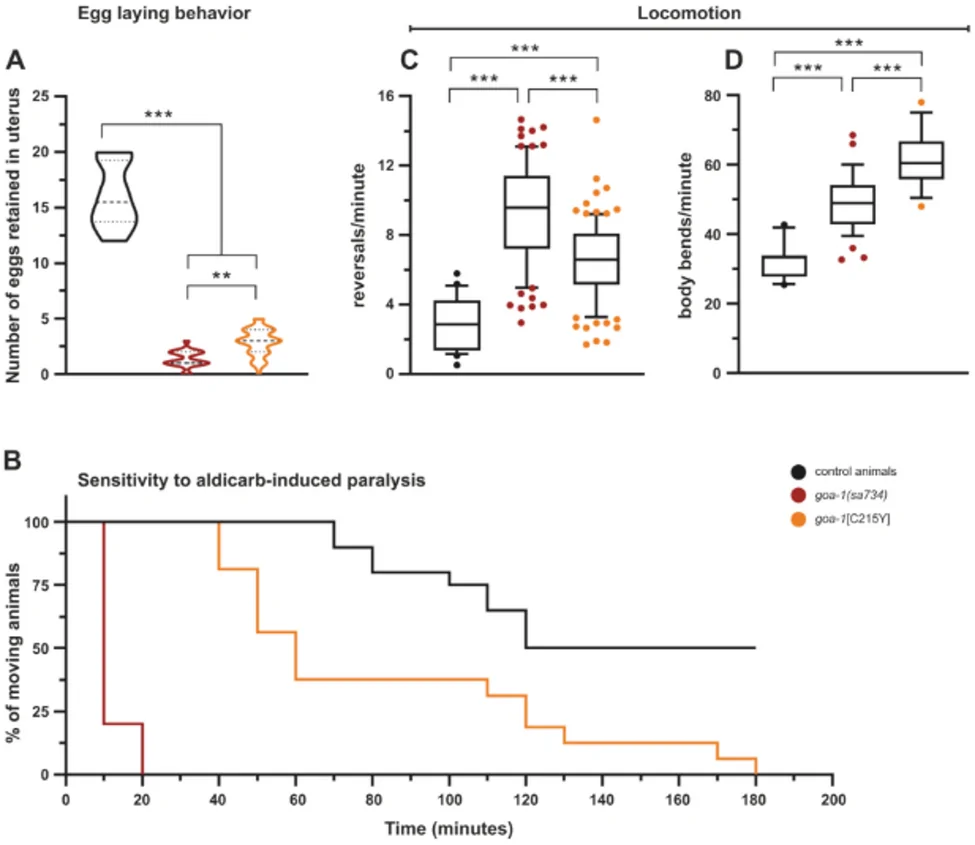
*goa-1*[C215Y] worms display hyperactive egg-laying and locomotion and increased ACh release at the*C. elegans*NMJ. **A** The egg-laying activity is quantified as number of eggs in the uterus of adult hermaphrodites during the exponential phase of egg-laying. Data are shown as violin plots, which display the distribution of individual values. The central line represents the median, while the upper and lower lines indicate the interquartile range. Statistical analysis was performed using one-way ANOVA followed by Bonferroni correction (**p < 0.002; ***p < 0.0001). **B***goa-1*[C215Y] mutants display hypersensitivity to aldicarb-induced paralysis (500 µM) compared to control animals (*p* < 0.0001 in all comparisons; log-rank test), suggesting increased ACh release by ventral cord motor neurons. **C**,** D** *goa-1*[C215Y] animals exhibit aberrant motor function as revealed by increased reversal rate (**C**) and number of body bends per minute (**D**) relative to controls (***p < 0.0001; one-way ANOVA followed by Bonferroni correction). The line inside the box represents the median; box edges mark the 25th and 75th percentiles, and whiskers extend to the 10th and 90th percentiles. Points beyond the whiskers are outliers. In A-D, twenty animals for each genotype were tested. The *goa-1(sa734)* null mutant was included as a reference in all experiments

We found that PHX7956 *goa-1*(*syb7956*) hermaphrodites carrying the C215Y variant (*goa-1*[C215Y]) phenocopied the behavior of a knock-out *goa-1* strain carrying the *goa-1*(*sa734*) null allele (c.154 C > T; p.Q52*), as well as the behavior of *goa-1* knock-in strains expressing variants associated with GNAO1 encephalopathy [11, 12]. In particular, *goa-1*[C215Y] adults retained significantly fewer eggs in utero during the peak period of egg-laying, averaging only 3 eggs compared to 15 eggs in control worms (Fig. 6A). This observation supports the hypothesis that C215Y leads to excessive neurotransmitter release, particularly serotonin [20], from HSN motor neurons that innervate vulval muscles, likely due to impaired Gαo-dependent inhibitory signaling in these cells. In addition, *goa-1*[C215Y] mutants exhibited increased sensitivity to aldicarb-induced paralysis compared to wild-type animals (Fig. 6B), although less severe than that observed in null mutants and other knock-in strains [11–12, 19]. These findings suggest enhanced ACh release by ventral cord motor neurons and its rapid accumulation at the *C. elegans* neuromuscular junction (NMJ). Finally, assessment of motor behavior through an automated tracking system [11, 27] revealed that *goa-1*[C215Y] mutants display hyperactive movement patterns, notably a higher frequency of spontaneous backward locomotion (“reversals”) relative to wild-type animals (Fig. 6C). This phenotype is consistent with increased glutamatergic signaling onto AVA interneurons, which plays a key role in this repetitive behavior [28]. Gene-edited animals also exhibited an increased number of body bends per minute (Fig. 6D), and moved faster than controls (means are 29.5, 75.2, and 67.8 μm/sec for N2, *goa-1*(*sa734*), and *goa-1*[C215Y], respectively; *p* < 10^− 6^, two-sample t-test). Collectively, these findings indicate that the C215Y substitution is a hypomorphic variant that partially impairs Gαo function in vivo across multiple neuronal contexts.

**Fig. 7:**
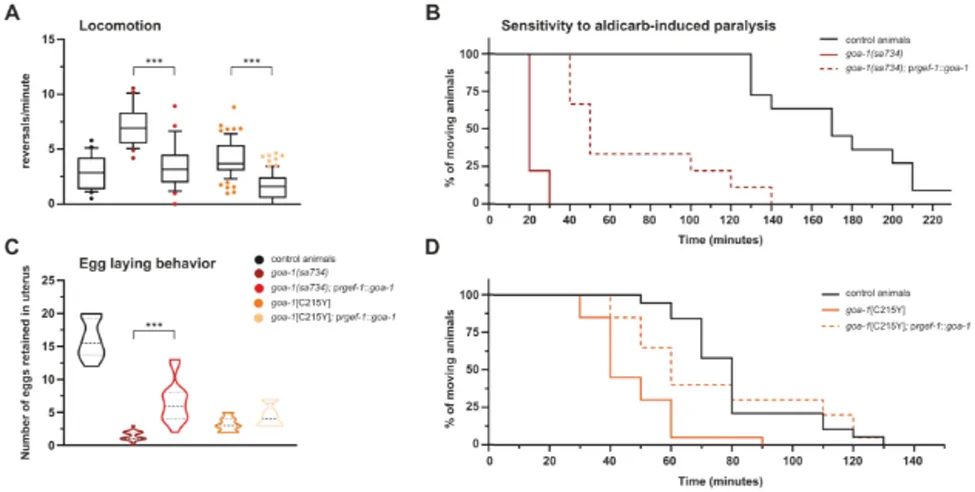
Neuronal expression of wild-type*goa-1*improves phenotypes of*goa-1*knockout and*goa-1*[C215Y] mutants.**A** Normal locomotion is restored in *goa-1(sa734)* and *goa-1*[C215Y] animals upon neuronal expression of wild-type *goa-1* driven by the *rgef-1* promoter (***p < 0.0001; one-way ANOVA with Bonferroni correction). **B***goa-1* transgene expression reduces aldicarb hypersensitivity in both mutant strains (*p* < 0.0001 for *goa-1(sa734)*;p*rgef-1*::*goa-1* compared to *goa-1(sa734)*; *p* < 0.002 for *goa-1*[C215Y]; p*rgef-1*::*goa-1* compared to *goa-1*[C215Y]; log-rank test). **C** *goa-1* overexpression ameliorates the hyperactive egg-laying phenotype in null mutants (***p < 0.0001; one-way ANOVA with Bonferroni correction), but not in *goa-1*[C215Y] animals, likely due to a dominant-negative behavior of this variant in HSN motor neurons. Twenty animals from three independent transgenic lines per genotype were analyzed. Violin and box plot parameters are as described in Fig. 6

To test the efficacy of gene supplementation, we generated transgenic lines overexpressing the wild-type *goa-1* allele in neurons of null and *goa-1*[C215Y] animals. Phenotypic analysis revealed a robust rescue of aberrant locomotion (reversal rate) in both strains (Fig. 7A) and a significant improvement in aldicarb sensitivity (Fig. 7B). In contrast, overexpression of wild-type *goa-1* was able to ameliorate the egg-laying phenotype only in null mutants, but not in *goa-1*[C215Y] worms (Fig. 7C), suggesting a dominant-negative behavior of this variant in HSN motor neurons.

### Gene supplementation is also effective in C. elegans strains carrying goa-1 mutations associated with severe phenotypes in humans

**Fig. 8:**
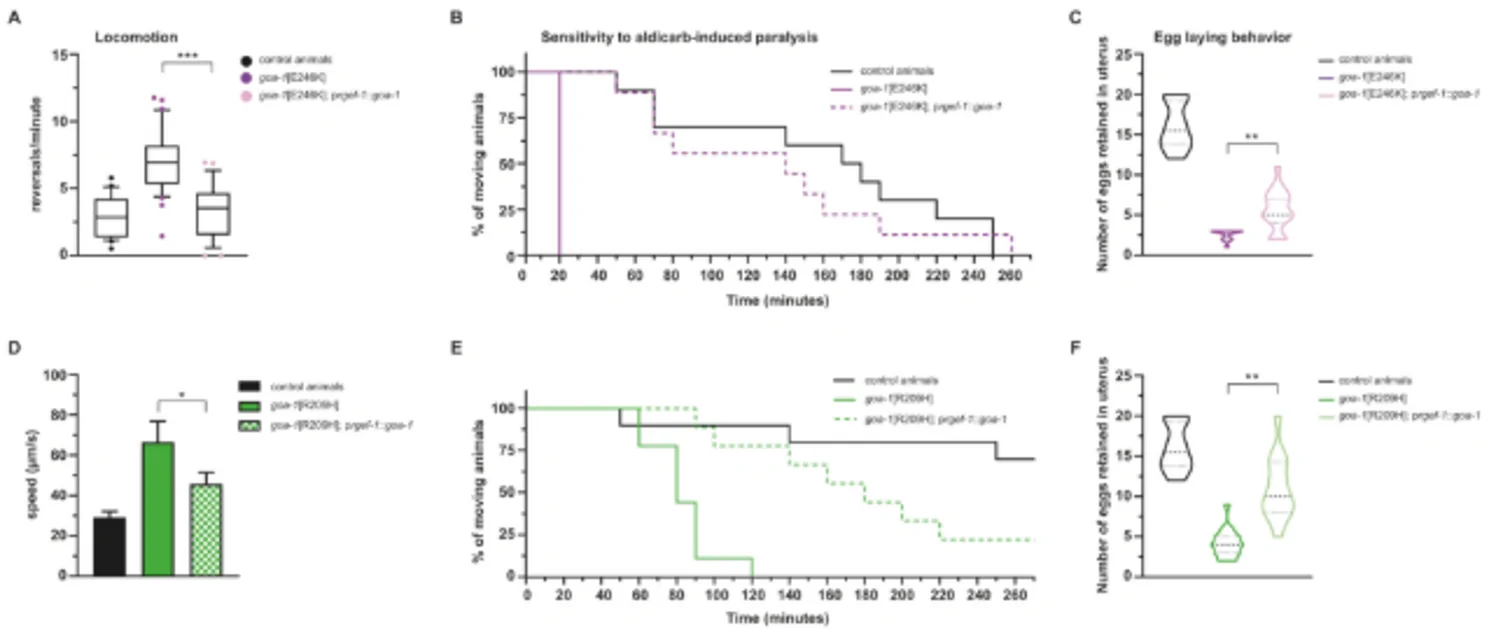
Neuronal expression of wild-type*goa-1*improves phenotypes of*goa-1*[R209H] and*goa-1*[E246K] mutants.**A**,** B** In *goa-1*[E246K] animals, neuronal expression of wild-type *goa-1* driven by the *rgef-1* promoter fully restores locomotion (***p < 0.0001; one-way ANOVA with Bonferroni correction) and aldicarb sensitivity (*p* < 0.0001 for *goa-1*[E246K]; p*rgef-1*::*goa-1 vs.*. *goa-1*[E246K]; log-rank test). **C** A partial rescue of the egg-laying phenotype is observed in HSN neurons (**p < 0.002; one-way ANOVA with Bonferroni correction). **D–F** In nematodes carrying the dominant-negative R209H mutation, neuronal overexpression of wild-type *goa-1* results in a milder but significant improvement across all phenotypes: locomotion speed (*p* < 0.05; one-way ANOVA with Bonferroni correction), aldicarb sensitivity (*p* < 0.0002 for *goa-1*[R209H]; p*rgef-1*::*goa-1 vs.*. *goa-1*[R209H]; log-rank test), and hyperactive egg-laying (**p < 0.002; one-way ANOVA with Bonferroni correction). Twenty animals from three independent transgenic lines per genotype were analyzed. Violin and box plot parameters are as described in Fig. 1. For the speed bar plot, data represent means ± SEM from multiple observations

To provide a proof-of-concept for gene supplementation as a broader therapeutic strategy independently of the specific mutation involved, we extended the overexpression analysis to knock-in strains carrying the R209H and E246K substitutions, which are among the most recurrent variants in clinical cases. Previous data showed that R209H behaves as a dominant-negative allele in cholinergic motor neurons and glutamatergic interneurons, but not in HSN motor neurons, whereas E246K does not exhibit a dominant-negative behavior in worms [12]. Overexpression of wild-type *goa-1* significantly improved all tested phenotypes in both *goa-1*[R209H] and *goa-1*[E246K] strains, including a modest but significant rescue of the egg-laying defect (Fig. 8). Since *goa-1*[R209H] mutants display increased locomotion speed despite only a mild increase in reversal frequency [12], we assessed locomotor speed rather than reversal rate for this variant.

Taken together, these results establish that neuronal expression of wild-type *goa-1* ameliorates the phenotype in knock-in *C. elegans* strains, even in the presence of variants acting in a dominant-negative manner. These findings underscore the therapeutic potential of gene supplementation and support its further evaluation in *Gnao1* mouse models and, ultimately, its translation to clinical application in patients.

## Discussion

*GNAO1* is a gene widely expressed in the nervous system, and it is important for neuronal differentiation [29]. GNAO1-related encephalopathy is a recently recognized rare pediatric neurological disorder primarily caused by missense mutations in the *GNAO1* gene. Current evidence implicates *GNAO1* mutations in dysregulation of cAMP signaling, impaired neurotransmitter release, and abnormal neuronal maturation, mechanisms elucidated through studies employing animal models of motor disorder phenotypes and molecular pathway analyses [30, 31]. It has been demonstrated that *GNAO1* silencing reduced neurite outgrowth during development [32, 33]. The early phenotypic manifestations of *GNAO1* variants in patients prompted us to test an AAV9-based gene therapy approach to restore wild-type Gαo expression in newborn *Gnao1*[C215Y]/+ mice. This approach leads to an increased expression of the protein in adult brain tissue and to the correction of behavioral and functional phenotypes in knock-in mutants. Previous studies demonstrated that Gnao1[C215Y]/+ mice exhibit a relatively mild phenotype, paralleling that described in patients, primarily late-onset hyperkinetic movement disorders [15]. By investigating the effects of the C215Y variants on neuronal excitability, we further showed that cortical neurons in heterozygous Gnao1[C215Y] mice exhibit a depolarized membrane potential, reduced cAMP-dependent, HCN channel-mediated inward currents (Ih), and smaller GABAB-mediated currents. These functional alterations were all attenuated or eliminated by AAV-mediated wild-type Gαo expression in newborn mice, indicating that this approach could correct or compensate for the effects of the endogenous mutant protein in neurons. In this study, using behavioral tests, we found that *Gnao1*[C215Y]/+ mice exhibit abnormal motor behavior and increased anxiety-like behavior. The gene therapy approach restored normal behavior; mice treated with AAV-wt-hGNAO1 showed reduced anxiety-like behavior and performed similarly to wild-type mice on the accelerating rotarod test. The increased latency to fall observed at t3 in C215Y/+ mutant could be explained by an increased anxiety-like behavior together with a more stable posture on the accelerating rotarod. Indeed, in many tests, anxious mice are less exploratory and more risk-avoidant, as in C215Y mutant mice. Another important factor to consider is that motor and coordination functions vary among sub-strains. C57BL/6J mice exhibited significantly longer latencies than C57BL/6 N and C57BL/6 C mice on rotarod [34].

HCN channels are critical regulators of neuronal excitability, rhythmic firing, and synaptic integration throughout cerebellar, thalamic, and limbic networks. Alterations of Ih have been shown to affect motor coordination and locomotor behavior through modulation of cerebellar and thalamocortical activity [35, 36]. Likewise, HCN channel dysfunction has been implicated in emotional regulation and anxiety-related phenotypes through its effects on neuronal excitability within hippocampal and cortical circuits [37]. Similarly, GABAB receptor signaling plays a key role in controlling neuronal excitability and neurotransmitter release in both motor and limbic pathways. In the cerebellum, GABAB receptor-mediated modulation contributes to the fine tuning of motor output, whereas alterations in GABAB-dependent signaling have been associated with anxiety-related behaviors and neuropsychiatric phenotypes [38, 39]. Therefore, although the present study does not directly address circuit-level mechanisms, the normalization of Ih and GABAB-dependent responses observed following treatment may contribute to the improvement of motor and behavioral phenotypes through restoration of network excitability within circuits known to regulate movement and emotional behaviors.

Together, these data indicate that gene supplementation during the early postnatal developmental stage could prevent C215Y-related phenotypic alterations in mice during young adulthood. We speculate that AAV-mediated delivery of wild-type *GNAO1* could restore the reduced pool of functional Gao by competing with interacting effector proteins. Recent studies explored the potential of AAV-RNAi vectors or allele-specific antisense oligonucleotides (ASOs) to suppress mutant *GNAO1* alleles, with the selective silencing of the pathogenic variant *GNAO1* c.607G > A (G203A) in vitro [40], in patient-derived neurons [41], and in vivo [18]. This approach specifically targets the mutant allele, while leaving the wild-type protein unaffected [42]. At difference with previous studies, we here performed a functional investigation of neurobiological alteration, including electrophysiological characterization of circuit-level dysfunction and its rescue, providing key advancement in the knowledge of the effects induced by gene replacement on neuronal function.

Further, unlike previous studies focusing on a single GNAO1 variant, the C. elegans platform enabled us to compare the response to gene supplementation across multiple pathogenic alleles and neuronal circuits, providing initial evidence that therapeutic efficacy may depend on the balance between loss-of-function and dominant-negative mechanisms operating in specific neuronal contexts.

*C. elegans* studies provide an evolutionarily conserved in vivo framework supporting the efficacy of gene supplementation. The *goa-1*[C215Y] knock-in strain phenocopied key features of impaired Gαo signaling, including hyperactive locomotion, heightened cholinergic sensitivity, and defective egg-laying, consistent with circuit-wide increases in neurotransmitter release. Neuronal overexpression of wild-type *goa-1* robustly rescued locomotor and synaptic phenotypes in both null and *goa-1*[C215Y] animals, demonstrating that increased Gαo dosage can effectively compensate for reduced signaling capacity. However, the persistence of egg-laying defects suggests dominant-negative effect in HSN motor neurons, revealing a context in which supplementation alone may be insufficient. Extending this approach to additional knock-in strains carrying clinically recurrent *GNAO1* variants (R209H and E246K) further supported the effectiveness of this strategy: overexpression of wild-type *goa-1* significantly improved locomotor, synaptic, and egg-laying phenotypes in both strains, including modest rescue of egg-laying despite the partially dominant-negative behavior of R209H.

Together, these findings indicate that neuronal gene supplementation can restore Gαo-dependent signaling across diverse cellular contexts, even for alleles with moderate dominant-negative properties, and underscore its promise as a broadly applicable therapeutic strategy, complementing our results in mouse models and supporting its development for *GNAO1* encephalopathy.

The partially different outcome of gene supplementation in *C. elegans* and mice should be interpreted in light of the cell-context-dependent mechanisms of *GNAO1* dysfunction. Previous studies in *C. elegans* showed that pathogenic *goa-1* variants exert variable combinations of loss-of-function and dominant-negative effects depending on the neuronal circuit examined [11, 12, 19]. In this framework, the C215Y variant appears to behave predominantly as a hypomorphic allele in AVA interneurons and ventral cord motor neurons, where neuronal overexpression of wild-type *goa-1* rescues reversal rate and significantly improves aldicarb hypersensitivity. Conversely, the lack of rescue of the egg-laying phenotype suggests that C215Y may exert a dominant-negative effect in HSN motor neurons, a cellular context in which increasing the wild-type *goa-1* dosage is insufficient to restore signaling.

This interpretation is consistent with cell-based and in vivo studies in mice indicating that *GNAO1* variants perturb Gαo signaling through variable combinations of loss-of-function and dominant-negative mechanisms in a neuron-type-specific manner [43]. The efficient rescue observed in *Gnao1*[C215Y]/+ mice does not exclude a dominant-negative component of C215Y, but rather suggests that the behavioral and electrophysiological phenotypes examined in this model are sufficiently compensated by increasing the pool of wild-type Gαo. Notably, the mouse analyses were focused on selected behavioral outputs and on cortical layer V pyramidal neurons, whereas the *C. elegans* egg-laying assay interrogates a distinct serotonergic motor-neuron circuit. Overall, these findings highlight that the efficacy of gene supplementation may depend on both the pathogenic variant and the neuronal population involved. From a translational perspective, this supports the therapeutic potential of *GNAO1* supplementation, particularly for variants and circuits in which reduced functional Gαo dosage is a major driver, while also highlighting the need to evaluate additional *GNAO1* alleles, neuronal subtypes, and clinically relevant endpoints to identify contexts in which allele-specific silencing or combined strategies may be required.

Future studies will be necessary to understand the molecular mechanisms underlying the observed effects and to evaluate whether this gene therapy approach could be applied to other *GNAO1* variants in mice or whether later treatments could result in phenotypic modifications.

## Supplementary Information

Below is the link to the electronic supplementary material.

Supplementary Material 1

## Data Availability

The datasets used and analysed during the current study available from the corresponding author on reasonable request.

## Abbreviations

AAV: adeno-associated virus
aCSF: artificial Cerebro Spinal Fluid
Aps: action potentials
C. elegans: Caenorhabditis elegans
DEE17: early-infantile severe developmental epileptic encephalopathy
GPi-DBS: Globus pallidus internus deep brain stimulation
Gαo: G protein subunit alpha O1
ICV: intracerebroventricular
Ih: inward currents
MD: Movement disorders
NGM: nematode growth medium
NMJ: neuromuscular junction
RPM: rotations per minute
