# Supplementary material for: Gene supplementation therapy restores motor deficits in Gnao1[C215Y] mice and proves broad efficacy in C. elegans model
Source: Acta Neuropathol Commun. 2026 Jul 21;14:217. doi: 10.1186/s40478-026-02389-6 (PMC13678979; doi:10.1186/s40478-026-02389-6)
Supplement: Supplementary file 1 [file 40478_2026_2389_MOESM1_ESM.docx]

**SUPPLEMENTARY INFORMATION FILE**

**Paper:**

**Gene supplementation therapy restores motor deficits in Gnao1[C215Y] mice and proves broad efficacy in *C. elegans* model**

**Cocozza et al**

**Figures**

**Figure S1.** Imaging of brain tissue from *Gnao1*[C215Y]/+(GFP) mice confirmed successful stereotaxic intracranial injection of AAV9.CAG.GFP in heterozygous animals. In these mice, the stereotaxic intracranial injection resulted in neuronal GFP expression throughout the whole brain. Panels A, C, D, and E show maximum-intensity projections of different coronal planes, while panels B and F show corresponding magnifications of the regions indicated in (A) and (E), respectively. All images were acquired using a CoolSNAP camera (Photometrics) mounted on an ECLIPSE Ti-S microscope (Nikon).

**Figure S2.** Western blot showing GNAO1 protein levels in brain tissue from wild-type (+/+), *Gnao1[C215Y]*/+/(GFP), and *Gnao1[C215Y]*/+/(h*GNAO1*) mice (N=3), with β-actin used as a loading control.
